# From Vision to Validation: The Journey of Tofersen in Superoxide Dismutase 1 Gene–Associated Amyotrophic Lateral Sclerosis

**DOI:** 10.1016/j.mayocpiqo.2026.100755

**Published:** 2026-09-07

**Authors:** Björn Oskarsson, Michael G. Heckman

**Affiliations:** Department of Neurology (B.O.), Division of Clinical Trials and Biostatistics, Department of Quantitative Health Sciences (M.G.H.), Mayo Clinic, Jacksonville, FL

Few stories in modern neurology illustrate the power of imagination and scientific innovation as the development of tofersen for superoxide dismutase 1 gene (*SOD1*)-associated amyotrophic lateral sclerosis (ALS). As an ALS clinician-scientist privileged to have cared for patients through the development period, the experience has been elating, even if it has not been without loss. Today, as real-world evidence accumulates from national cohorts and international treatment programs, the story of tofersen is told in a new and perhaps even more consequential chapter, helping a growing number of people.^1^

What began as a bold idea championed by a determined clinician, who persuaded visionary scientists and engaged expert drug developers, has now evolved into one of the most important precision medicine achievements in neurodegenerative disease. This story starts with Richard Smith, followed quickly by Don Cleveland and Frank Bennett and the team at Ionis through a series of trials led by Timothy Miller, all of whose roles have been transformative of the landscape of *SOD1*-ALS treatment. The vision was simple but revolutionary: if a pathogenic vaiant causes disease, perhaps directly targeting that pathogenic vaiant with an intrathecal antisense oligonucleotide (ASO) could alter its course.

At the time, such thinking was far from mainstream. Yet, advances in molecular biology were beginning to reveal opportunities for intervention. The first genetic cause of ALS was identified in the *SOD1*, responsible for 2% of US ALS cases.^2^ Years of research demonstrated that pathogenic vaiant SOD1 proteins exerted toxic effects on motor neurons while knockouts were viable, making the gene an attractive gene silencing target.

The emergence of ASO technology offered a potential solution.^3^ These short synthetic strands of nucleic acid could be designed to bind specific mRNA sequences, tagging them for degradation and thereby reducing production of harmful proteins. The resulting ASO therapy was designed to reduce production of SOD1 protein directly within the central nervous system. The concept represented a shift in ALS therapeutics. Rather than attempting to broadly protect neurons from damage, the ASO sought to address one root cause of disease.

Early clinical studies generated cautious optimism.^4^^,^^5^ Investigators reported that tofersen could considerably reduce SOD1 protein concentrations in cerebrospinal fluid.^6^ As importantly, treatment was associated with substantial reductions in neurofilament light chain (NfL), a biomarker that reflects neuroaxonal injury and has emerged as the most important indicator of disease activity in ALS.^7^ These biological signals suggested that the therapy was engaging its intended target and altering disease biology. Yet the path forward was not straightforward.

The pivotal phase 3 A Study to Evaluate Efficacy, Safety, Tolerability, Pharmacokinetics and Pharmacodynamics of Tofersen in *SOD1*-ALS (VALOR) trial delivered results that were both encouraging and controversial.^6^ In this study, there was no statistically significant difference between the tofersen and placebo groups regarding the primary outcome of change in ALS Functional Rating Scale Revised score from baseline to week 28, where the primary analysis involved the faster-progression subgroup, a finding that immediately generated debate within the ALS community. Critics questioned whether the biological effects observed with tofersen would ultimately translate into meaningful clinical benefit. Supporters argued that ALS progression, especially in genetically heterogeneous populations, may require longer observation periods to reveal therapeutic effects and that the clinical outcome measures lack sensitivity.

Subsequent data strengthened the latter position. Participants receiving earlier treatment reported sustained reductions in NfL and appeared to experience slower functional decline over time.^8^ The biological signal remained remarkably consistent: lowering SOD1 led to lower NfL, indicating reduced neuronal injury.

This evolving evidence base persuaded regulators. In 2023, the United States Food and Drug Administration granted accelerated approval, recognizing the importance of NfL reduction as a reasonably likely surrogate marker of clinical benefit. European approval followed in 2024. For the first time, patients with *SOD1*-ALS had access to a therapy specifically designed to target the genetic cause of their disease. However, regulatory approval did not mark the end of the scientific journey. Rather, it initiated an equally important phase: understanding how tofersen performs in routine clinical practice.

Randomized controlled trials remain the gold standard for establishing efficacy, but they include carefully selected patient populations and relatively short observation periods. Though real-world, non-randomized studies are obviously limited by the biases and potential for confounding introduced by the lack of randomization, such studies importantly provide a broader perspective, capturing the diversity, complexity, and variability of patients encountered in clinical practice. Recent real-world studies have begun to offer exactly that perspective.

Among the most relevant contributions is the French FORSLA study, a nationwide multicenter investigation conducted through the Filière de santé SLA et Maladies du Neurone moteur network, published in this issue of *Mayo Clinic Proceedings: Innovations, Quality, & Outcomes*.^1^ This retrospective analysis evaluated 46 patients treated with tofersen across 19 ALS expert centers and compared outcomes both within treated individuals and against a historical cohort of patients receiving standard care and yielded additional promising findings regarding the use of tofersen in the treatment of *SOD1*-ALS. Specifically, patients receiving tofersen reported a substantial slowing of disease progression, where mean ALSFRS-R progression rates decreased from 0.53 points per month before treatment to 0.22 points per month after 12 months of therapy. At the same time, NfL fell dramatically, dropping from ∼89 pg/mL at baseline to ∼29 pg/mL after 1 year of treatment. These changes mirror the biological effects observed in earlier clinical trials and reinforce the growing confidence that tofersen is altering the disease process itself.

Also of interest were comparisons of survival between the tofersen cohort and historical controls. Though it is important to consider the possibility that these comparisons could be influenced to some extent by unmeasured confounding variables/biases in this nonrandomized setting, in propensity-score-matched analyses, patients treated with tofersen experienced significantly longer survival than comparable historical controls. Specifically, the adjusted hazard ratio of 0.34, where 95% confidence limits ranged from 0.12 to 0.91, suggests that tofersen is associated with at the very least a small improvement in survival and possibly a 3 to 8-fold improvement in this key outcome measure. The relatively small sample size of these analyses (N=39 matched pairs in propensity score analysis) limits the strength of the conclusions that can be made, but nonetheless, these findings are promising and highlight the need for further study (and meta-analytical approaches) of tofersen in ALS.

The French experience aligns with reports from Germany and other early-access programs.^9^^,^^10^ Across multiple health care systems, investigators are observing similar patterns: reductions in NfL, stabilization of functional decline, and signals suggesting improved long-term outcomes. The consistency of these observations strengthens confidence that the benefits of tofersen extend beyond the controlled environment of clinical trials.

Of importance, these studies also provide practical insights into treatment implementation. Clinicians have gained experience managing adverse events associated with intrathecal administration, including inflammatory reactions. Although serious adverse events occur, they appear to be rare and generally manageable. Such information is invaluable as treatment expands into broader clinical practice.

The broader relevance of tofersen extends beyond *SOD1*-ALS itself. The therapy represents a proof of principle for precision medicine in neurodegeneration. For decades, neurological diseases were largely treated according to clinical symptoms rather than molecular mechanisms. Tofersen demonstrates that targeting a specific genetic cause can produce measurable biological and clinical effects. This achievement has energized efforts to develop similar approaches for other genetic forms of ALS and other neurodegenerative diseases.

Today, the story of tofersen is a new paradigm for ALS treatment. The accumulating body of evidence—from laboratory studies to randomized trials, open-label extensions, and now large-scale real-world cohorts—points in a consistent direction. Although important questions remain regarding optimal timing, maybe even presymptomatic treatment, inflammatory side effects, long-term outcomes, and patient selection factors, including specific genotypes, the trajectory of evidence continues to strengthen.

For patients with *SOD1*-ALS, tofersen offers something that was only imaginable: a potent therapy designed specifically for the biology of their disease. For the ALS community at large, it provides proof that treatments can succeed.

The journey that began with one person’s curiosity has evolved into one of the most important success stories in modern neurodegenerative medicine. Real-world evidence is now validating what many hoped—that reducing SOD1 can translate into meaningful benefits for patients. In doing so, tofersen is not only changing the course of a rare genetic form of ALS; it is reshaping expectations for the future of precision therapies across neurology.

## Potential Competing Interests

Dr Oskarsson has received grant funding from Biogen in the past. The authors report no other conflicts of interest.

## Declaration of Generative AI and AI-Assisted Technologies in the Writing Process

During the preparation of this work, the authors used ChatGPT to expand the conceptual draft. After using this tool, the author reviewed and edited the content as needed and take full responsibility for the publication's content.
